# Open access publishing in health and social care simulation research – Advances in Simulation

**DOI:** 10.1186/s41077-015-0002-x

**Published:** 2016-01-11

**Authors:** Debra Nestel

**Affiliations:** Faculty of Medicine, Nursing & Health Sciences, Monash University, Clayton, 3800 Australia

Although simulators have been used in healthcare education for hundreds of years, it is really only in the last thirty years that research *with* and *about* simulation has grown, and this growth has been exponential. The research has been diverse with respect to intent, simulation modality and context. It has been descriptive, experimental, evaluative, explanatory and exploratory, meaning the methodologies and methods have drawn from quantitative, qualitative and mixed methods research approaches. Researchers and their audiences are also diverse and include simulation practitioners, health and social care professionals and educators, psychologists, sociologists, biomedical scientists, engineers, information technologists, economists, programme evaluators, policy makers and others. Within this context *Advances in Simulation* takes its place as an open access scholarly publication at BioMed Central. *Advances in Simulation* will provide a forum for all to share scholarly research, ideas, policies and practices that advance the uses and theories of simulation in the health and social care community.

Our journal fulfils an aspiration of the Executive of the Society in Europe for Simulation Applied to Medicine (SESAM), under the leadership of immediate Past President, Dr Ralf Krage and current President, Prof Antoine Tesniere. As Editor in Chief, I am granted editorial independence and together with the Senior Editors, Dr Peter Dieckmann (Denmark), Prof Tanja Manser (Germany) and Prof Ryan Brydges (Canada), we are charged with the responsibility of shaping the forum offered by *Advances in Simulation*. Our Editorial Board [1] comprises individuals representing the breadth of simulation research, development, policy and practice experience described above. We have a multi-layered Editorial Board with Associate Editors, Editors and an International Advisory group.

We consider quality to be measured by more than the published article. We will aim to provide authors who submit manuscripts to *Advances in Simulation* with an experience that values them as members of the health and social care simulation community. We will offer thoughtful and fair peer review – only individuals with highly relevant expertise will be invited to review. We will also strive for timeliness in the review process and will rely on our many reviewers to achieve this. We will operate a continuous publication model for articles that are successful in the peer-review process, with articles published quickly following acceptance.

We will consider manuscripts that apply to all health and social care professions and draw on all sciences, from explorations of cognitive mechanisms to societal impacts. We will publish basic and applied research, reviews, commentaries, innovations, debates, methodology articles and editorials [2]. While our guidelines honour conventional research reporting, we will also welcome alternative submissions that give our community members the opportunity to experiment with how they present data and findings.

In 2010, Regehr suggested the need to re-orient two of the dominant discourses in health professions’ education research: (i) from the imperative of proof to one of understanding, and (ii) from the imperative of simplicity to one of representing complexity well [3]. And while the scope of *Advances in Simulation* is much wider than education, his words resonate with our intent to value research seeking understanding *with* and *about* simulation. Moreover, as our community asks more complex questions, and studies more complex uses of simulation technologies, researchers have a responsibility to thoughtfully align research paradigms with hypotheses and research questions. Our intent is to provide a forum for all discourses, with a particular emphasis on researchers who demonstrate a clear fit of paradigm to scholarly purpose.

Over the next two years we have commissioned several articles addressing issues the Editorial Board and members of SESAM have identified as important and relevant for their practices such as conducting economic evaluations, the potential of serious games, the role of simulation in career transitions and more.

The *open access* publishing model of *Advances in Simulation* enables articles to be freely available and universally accessible. This has obvious benefits. In open access publishing, authors retain copyright of their work. Articles will also be archived in PubMed Central and other freely available full-text repositories. This archiving complies with policies of major funding bodies. Authors pay the article processing charge, which covers the cost of the publication process enabling readers to have free access.

Our community is experiencing an exciting era in health and social care simulation research and practice. We hope that you will contribute to *Advances in Simulation* through submissions, reading and sharing published articles, serving as a reviewer, and proposing future topics for this forum.

Debra Nestel, Editor in Chief, *Advances in Simulation.*
